# Observation of unpaired substrate DNA in the flap endonuclease-1 active site

**DOI:** 10.1093/nar/gkt737

**Published:** 2013-08-23

**Authors:** L. David Finger, Nikesh Patel, Amanda Beddows, Long Ma, Jack C. Exell, Emma Jardine, Anita C. Jones, Jane A. Grasby

**Affiliations:** ^1^Department of Chemistry, Centre for Chemical Biology, Krebs Institute, University of Sheffield, Sheffield, S3 7HF, UK and ^2^EaStCHEM School of Chemistry and Collaborative Optical Spectroscopy, Micromanipulation and Imaging Centre, The University of Edinburgh, West Mains Road, Edinburgh EH9 3JJ, UK

## Abstract

The structure- and strand-specific phosphodiesterase flap endonuclease-1 (FEN1), the prototypical 5′-nuclease, catalyzes the essential removal of 5′-single-stranded flaps during replication and repair. FEN1 achieves this by selectively catalyzing hydrolysis one nucleotide into the duplex region of substrates, always targeting the 5′-strand. This specificity is proposed to arise by unpairing the 5′-end of duplex to permit the scissile phosphate diester to contact catalytic divalent metal ions. Providing the first direct evidence for this, we detected changes induced by human FEN1 (hFEN1) in the low-energy CD spectra and fluorescence lifetimes of 2-aminopurine in substrates and products that were indicative of unpairing. Divalent metal ions were essential for unpairing. However, although 5′-nuclease superfamily-conserved active-site residues K93 and R100 were required to produce unpaired product, they were not necessary to unpair substrates. Nevertheless, a unique arrangement of protein residues around the unpaired DNA was detected only with wild-type protein, suggesting a cooperative assembly of active-site residues that may be triggered by unpaired DNA. The general principles of FEN1 strand and reaction-site selection, which depend on the ability of juxtaposed divalent metal ions to unpair the end of duplex DNA, may also apply more widely to other structure- and strand-specific nucleases.

## INTRODUCTION

Structure-specific phosphodiesterases play essential cellular roles by recognizing and acting on aberrant nucleic acid structures. Nucleic acid structures that require processing in this way include bubbles, flaps, nicks, gaps and four-way DNA junctions, which occur as intermediates during DNA replication, repair and recombination. To return to the duplex state and thereby restore the genome, the ends of intact duplexes contained within these more complex structures must undergo site-selective strand-specific phosphate diester hydrolyses. In line with this key role in maintaining genome integrity, defects in structure-sensing nucleases lead to a range of diseases, including cancer (1,2).

Exemplary strand-specific duplex-targeting phosphodiesterases are the flap endonuclease (FEN)-family nucleases, also known as the 5′-nuclease superfamily, whose activities involve processing a range of aberrant nucleic acid substrates (3). FEN1 is an essential component of the lagging-strand DNA replication, long-patch base excision repair and ribonucleotide excision repair pathways. FEN1 removes 5′-single-stranded protrusions, known as flaps, that are formed during DNA polymerase-catalyzed strand displacement synthesis. Other protein-sequence-related 5′-nucleases include EXO1, the 5′-nuclease that catalyzes resection of duplex, nicked and gapped DNAs during mismatch and double-strand break repair, and XPG, the 5′-nuclease of nucleotide excision repair that targets DNA bubbles. Another family member, GEN1, processes DNA four-way (Holliday) junctions. Although members of the 5′-nuclease family act on a diverse range of substrates, one feature of their reactions is universal: all FEN family nucleases catalyze hydrolysis one nucleotide into a double-stranded region of their more complex target DNAs. Moreover, this reaction always occurs on the 5′-strand of the duplex abutting the nucleic acid junction.

**Figure 1.**
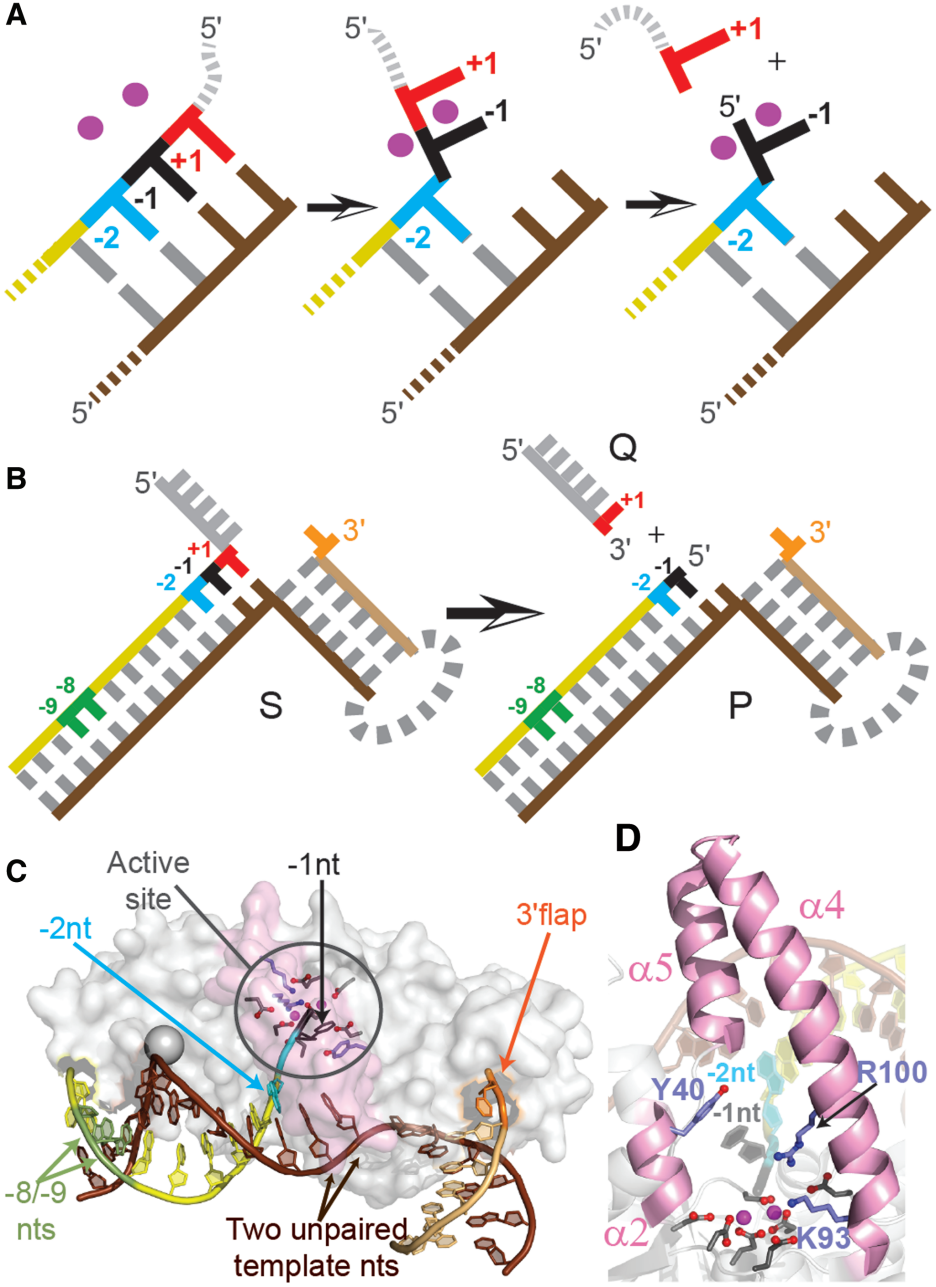
Schematic of proposed 5′-nuclease double nucleotide unpairing mechanism, reaction and supporting hFEN1-product structures. (**A**) Proposed unpairing of duplex ends that places the scissile phosphate diester in contact with active-site divalent ions (magenta). (**B**) The reaction of a static double-flap substrate (S) catalyzed by hFEN1 generates a 5′-phosphorylated product (P) and a single-stranded product (Q). Hydrolysis occurs between the +1 and −1 nucleotides (nts) as shown. (**C**) Structure of the hFEN1-product complex (3Q8K) highlighting two unpaired nucleotides of the template strand (brown), a single unpaired nt (−1 nt, black) of the CF strand (yellow) positioned on active-site metal ions (magenta), the 3′-flap nt (orange), the −2 nt (cyan), and −8 and −9 nts (green). (Product Q was not observed.) Protein is shown transparent with α4-α5 in pink. (**D**) The active site of hFEN1-P (colors as C). Side chains of superfamily conserved carboxylate residues (gray), K93, R100 and semi-conserved Y40 (light blue) are illustrated as sticks.

This precise reaction site selection is critical to 5′-nuclease action. For example, FEN1 is tasked to create ligatable nicked-DNA products so as to prevent erroneous genome-endangering incision that would require the subsequent action of additional repair pathways. The reaction-site-specificity of superfamily proteins has been explained by a novel double nucleotide unpairing mechanism that only allows a specific phosphate diester at the 5′-end of the duplex to contact the catalytic active-site divalent metal ions (Figure 1A) (3–7). This mechanism was initially inferred from biochemical studies and X-ray structures of a FEN-family protein with substrate, where the scissile phosphate to undergo reaction was seen bound in front of, not within, the active site (4,5,8).

More recently, structures of human FEN1 (hFEN1) and human EXO1 (hEXO1) bound to product DNAs provided support for this mechanism (6,7). In product complexes, hydrolyzed 5′-phosphate monoester was directly coordinated to two metal ions within the active site; this conformation required the terminal nucleotide of product to be extra-helical. Recently, we showed that interstrand cross-linking of the nucleobases proposed to unpair prevented hFEN1 reaction within the duplex DNA, lending further support for the double nucleotide unpairing mechanism (9). However, although a double nucleotide unpaired conformation of substrate is implied by biochemical and structural data, it has yet to be observed. Here, we study the 5′-nuclease unpairing mechanism by monitoring local DNA conformational changes using low-energy (>300 nm) CD and fluorescence decays of substrate and product containing the nucleobase analogue 2-aminopurine (2AP), which can replace adenine in DNA without significant structural perturbation (10–15). These data provide the first observations of unpaired conformations in DNA substrates bound to hFEN1 in the presence of catalytically inert active-site metal ions and define the role of amino acid residues and divalent metal ion cofactors in this process.

## MATERIALS AND METHODS

### DNA constructs

DNA oligonucleotides including those containing site-specific 2AP substitutions were purchased with purification by high performance liquid chromatography from DNA Technology A/S. DNA concentrations were determined by ultraviolet absorbance at 260 nm (20°C). Heteroduplexes were formed by heating the appropriate 2AP ssDNA with the complementary template in a 1:1.1 ratio at 80°C for 5 min in 50 mM Tris–HCl (pH 7.5) and 100 mM KCl with subsequent cooling to room temperature. Sequences are given in Supplementary Figure S1.

### Enzymes

hFEN1 and mutants were over-expressed and purified as described (6).

### CD spectroscopy

Samples containing 10 µM of the indicated DNA construct, 50 mM Tris–HCl (pH 7.5), 100 mM KCl, 1 mM dithiothreitol (DTT) and, where appropriate, 11–15 µM protein and either 10 mM CaCl_2_ or 10 mM CaCl_2_ + 25 mM EDTA were prepared with subsequent acquisition of CD spectra (300–480 nm) at 20°C using a JASCO J-810 CD spectrophotometer. CD spectra are an average of five scans recorded in 0.5 nm steps (0.5 s response time) that were baseline corrected using spectra recorded on samples containing the same components, but lacking DNA. The baseline-subtracted spectra were then corrected by smoothing using the Means–Movement option with a convolution width of five in the JASCO Spectra Analysis software (version 1.53.07). The CD spectra were plotted as Δ*ε* per mol 2AP residue versus wavelength. Each measurement was independently repeated (typically in triplicate) and gave identical results.

### Time-resolved fluorescence spectroscopy

Samples contained 10 µM of the indicated DNA construct, 50 mM 4-(2-hydroxyethyl)-1-piperazineethanesulfonic acid (HEPES) (pH 7.5), 100 mM KCl, 1 mM dithiothreitol (DTT) 10 mM CaCl_2_ and, where appropriate, 10 µM protein and were monitored at 20°C. Fluorescence decays were measured using the technique of time-correlated single photon counting, following the same procedure reported previously (13). The excitation source was the third harmonic of the pulse-picked output of a Ti-Sapphire femtosecond laser system (Coherent, 10 W Verdi and Mira Ti-Sapphire), consisting of pulses of ∼200 fs duration at a repetition rate of 4.75 MHz and a wavelength of 315 nm. Fluorescence decays were measured in an Edinburgh Instruments spectrometer equipped with TCC900 photon counting electronics. The instrument response of the system was ∼80 ps full-width at half-maximum. Fluorescence decay curves were analyzed using a standard iterative reconvolution method, assuming a multi-exponential decay function. Decays were collected at three emission wavelengths (370, 380 and 390 nm) and analyzed globally using Edinburgh Instruments ‘FAST’ software (i.e. they were fitted simultaneously) with lifetimes, τ_i_, as common parameters. The quality of fit was judged on the basis of the reduced chi-square statistic and the randomness of residuals. Results from repeat measurements (typically 3) were in good agreement, and the uncertainties in reported values of lifetimes and A factors are ≤10%.

## RESULTS

### Substrate design

The structure that is specifically recognized by eukaryotic FEN1 proteins is a two-way junction known as a 5′-3′-double flap that has a 5′-flap of any length (including none) and a single nucleotide 3′-flap (Figure 1B). When FEN1-catalyzed hydrolysis occurs one nucleotide into the 5′-duplex (between the +1 and −1 residues in the 5′-flap strand), the resulting product is nicked DNA that can be immediately joined by DNA ligase. Nicked DNA is the result of FEN1 action because both 5′-flaps and 3′-flaps are complementary to the template strand. However, such equilibrating 5′-3′-double flaps can adopt a variety of structures complicating analyses; ambiguity can be prevented by using non-complementary (i.e. static) double-flap constructs that undergo reactions with the same specificity as their equilibrating counterparts (16,17). To monitor conformational changes in hFEN1 substrates, using low-energy CD and time-resolved fluorescence spectroscopies, we designed static, double-flap substrates (S) and the corresponding product (P) from two oligomers, where the flap (F) or cleaved-flap (CF) strand contained one or more 2AP residues (Figure 1B).

### Spectroscopic analyses

Initially, we used time-resolved fluorescence of 2AP-containing DNA constructs to explore local conformational distortion induced by hFEN1 binding. Although these experiments proved informative, the interpretation of the 2AP decay parameters was complicated by the effect of interaction of 2AP with the Y40 residue of hFEN1 as well as with neighboring bases in the duplex. We, therefore, turned to low-energy CD, which reports only on the extent of stacking interaction between two adjacent 2APs strategically positioned within the duplex and gives a clear qualitative indication of local distortion. The low-energy CD results will be reported first, before considering the more-detailed information provided by time-resolved fluorescence.

### Product unpairing monitored by low-energy CD

To establish a signal for altered nucleic acid conformations in hFEN1-DNA complexes, we began by investigating the low-energy CD spectra of free and bound product. In X-ray structures of hFEN1-product complexes, a single unpaired nucleotide in the −1 position is unstacked from its nearest neighbor, the −2 nucleotide (Figure 1A and C) (6). A product containing a 2AP dimer, P_−__1__−__2_, (numbering denotes the positioning of 2AP modifications), was created to monitor any changes in relative positions of the −1 and −2 nucleobases by low-energy CD (Figures 1B; Supplementary Figure S1). Adjacent 2APs dimers form an exciton-pair with two singly excited electronic transitions of unequal energies and oscillator strengths (absorption intensities). The energies and intensities of the electronic transitions are dependent on the respective orientation of the electronic transition dipoles of each 2AP in the dimer pair, and therefore, spectroscopic changes reflect the local DNA conformation. These changes are most easily observed in CD spectra where a bisignate signal is observed for exciton-coupled pairs. In our case, only the red-shifted portion of the bisignate signal is observed due to interference from the DNA absorption below 300 nm. Moreover, this red-shifted signal reaches is maximum at 320−330 nm, a region of the CD spectra where protein and unmodified DNA are transparent (10). Thus, 2AP-dimer containing DNAs can be used to study conformational changes, such as unpairing, in DNA and DNA-protein complexes (10).

**Figure 2.**
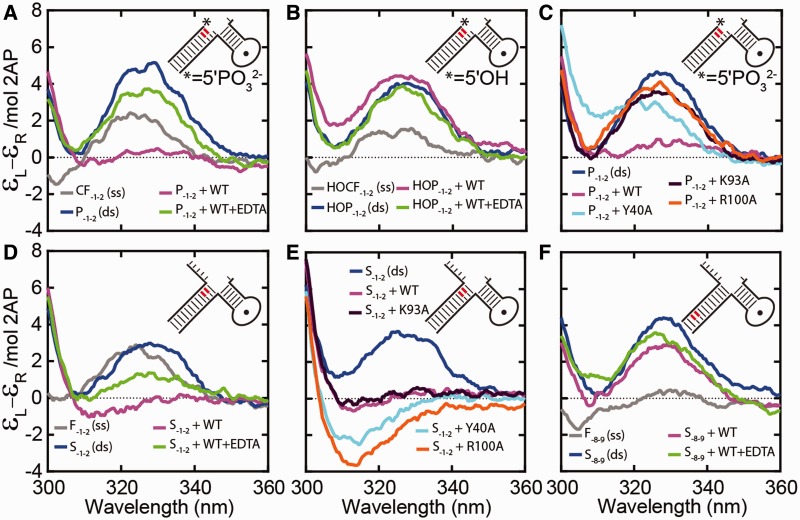
Low-energy CD spectra of 2AP-containing DNAs and hFEN1 WT- and mutant-DNA complexes. DNA constructs are illustrated schematically (red 2AP) with sequences shown in Supplementary Figure S1. (**A**) A dramatic divalent metal ion dependent reduction in 2AP exciton coupling signal occurred when product P_−1−2_ was bound to hFEN1, indicative of product unpairing. Unbound P_−1−2_ (blue), the corresponding single strand (CF_−1−2_, gray) and P_−1−2_ bound to hFEN1 (magenta) all in Ca^2+^ containing buffer. P_−1−2_ bound to hFEN1 in buffer containing 25 mM EDTA (green). (**B**) When product is not 5′-phosphorylated, (HO-P_−1−2_), there was no reduction in 2AP exciton coupling on addition of hFEN1- Ca^2+^. Unbound HO-P_−1−2_ (blue), the corresponding 2AP single strand (HO-CF_−1−2_, gray) and HO-P_−1−2_ bound to hFEN1 (magenta) all in Ca^2+^ containing buffer. HO-P_−1−2_ bound to hFEN1 in buffer containing 25 mM EDTA (green). (**C**) The presence of 5′-nuclease superfamily conserved K93 and R100 were required to reduce exciton coupling of P_−1−2_. Unbound P_−1−2_ (blue), P_−1−2_ bound to WT hFEN1 (magenta), Y40A (cyan), K93A (purple) and R100A (orange) in Ca^2+^ containing buffer. See Supplementary Figure S3A for spectra in EDTA buffer. (**D**) A similar dramatic divalent metal ion-dependent reduction in 2AP exciton coupling signal occurred when substrate S_−1−2_ was bound to hFEN1, indicative of substrate unpairing. Unbound S_−1−2_ (blue), the corresponding 2AP single strand (F_−1−2_, gray), and S_−1−2_ bound to WT hFEN1 (magenta) in Ca^2+^ buffer. S_−1−2_ bound to hFEN1 in buffer containing 25 mM EDTA (green). (**E**) The presence of 5′-nuclease superfamily conserved K93, R100 and Y40 were not required to reduce exciton coupling of S_−1−2_. Unbound S_−1−2_ (blue) and S_−1−2_ bound to WT hFEN1 (magneta), Y40A (cyan), K93A (purple), R100A (orange) all in Ca^2+^ containing buffer. (**F**) No changes in low energy CD signal occurred on complexation when 2APs were located away from the region of the substrate proposed to unpair. Unbound S_−8−9_ (blue), the corresponding single strand (F_−8−9_, gray) and S_−8−9_ bound to hFEN1 (magenta) all in Ca^2+^ containing buffer. S_−8−9_ bound to hFEN1 in buffer containing 25 mM EDTA (green). Each measurement was independently repeated and gave equivalent results.

The amplitude of the signal for the exciton-coupled 2APs contained within the single-stranded (ss) DNA (CF_−__1__−__2_) was considerably enhanced and red-shifted when the double-stranded (ds) P_−__1__−__2_ construct was formed (Figure 2A) (10). On formation of the hFEN1-P_−__1__−__2_ complex in the absence of divalent metal ions (presence of EDTA), only a small (25% *cf.* product in EDTA) decrease in the CD signal at 330 nm was observed, despite electrophoretic mobility shift assay results showing that at least 95% of the product was bound (Supplementary Figure S2A). Thus, only a minor alteration of the respective orientations of the two 2AP nucleobases occurs on binding hFEN1 without active-site divalent metals. In contrast, addition of hFEN1 in the presence of catalytically inert Ca^2+^ ions dramatically reduced the amplitude of the signal at 330 nm (83% cf*.* product in Ca^2+^ buffer) to near zero, implying a significant change of the relative orientation of the two 2AP residues (Figure 2A). However, when product lacked its 5′-phosphate monoester (HO-P_−__1__−__2_), negligible changes in the CD spectra occurred on addition of protein, regardless of the presence of Ca^2+^ ions (Figure 2B). Notably, both P_−__1__−__2_ and HO-P_−__1__−__2_ in complex with hFEN1 produced the identical signals in EDTA. Thus, hFEN1-induced conformational change of the 3′-product (P) DNA requires both divalent metal ions and the 5′-phosphate monoester, consistent with the extra-helical conformation of the −1 nucleotide observed in hFEN1 structures, where phosphate monoester is directly bound to active-site metal ions (Figure 1C and D).

In hFEN1-unpaired product structures, the metal-bound 5′-phosphate monoester is contacted by superfamily conserved residues K93 and R100 and the unpaired −1 base is stacked with Y40 (Figure 1D) (6). When P_−__1__−__2_ complexes were formed in the presence of Ca^2+^ ions with K93A and R100A mutated FENs, no evidence for conformational change about the −1 and −2 nt was observed, when compared with similar complexes formed in the presence of EDTA (Figures 2C; Supplementary FigureS3A). In contrast, Y40A complexes displayed a decrease in signal at 330 nm (51% cf. product in Ca^2+^ buffer) with Ca^2+^ ions (Figure 2C), but this was not as dramatic as that observed for wild-type (WT) protein complex (Figure 2A).

### Substrate unpairing monitored by low-energy CD

To investigate substrate unpairing in hFEN1 complexes, CD spectra of the bound double-flap substrate S_−__1__−__2_ were determined ± catalytically inert Ca^2+^ ions. Like the product complex, a Ca^2+^-dependent reduction in signal (98% cf*.* substrate duplex in Ca^2+^ buffer) was observed in S_−__1__−__2_ complexes (Figure 2D). Thus, both substrate and product undergo conformational changes that have a similar effect on the relative positions of the −1 and −2 2APs in the presence of hFEN1-Ca^2+^. This decrease in the magnitude of exciton-coupling (83% cf. substrate in Ca^2+^ buffer) was also observed in the presence of K93A-mutated hFEN1 and Ca^2+^ ions (Figure 2E), showing that, unlike product, substrate conformational change with respect to the −1 and −2 nucleotides is not dependent on this residue. Mutated hFEN1s, R100A and Y40A, also produced a dramatic change in the CD spectra, with a deep minimum present at 315 nm, in the presence of Ca^2+^ ions, implying that reorientation of the −1 and −2 nt also occurs in these complexes, but the precise juxtaposition or the partition between paired and unpaired forms differs from those in the WT and K93A complexes (Figure 2E).

In all cases, addition of EDTA to the Ca^2+^-enzyme-substrate complexes, which returned the signal to that of a complex formed in EDTA alone and allowed subsequent analysis of the samples by high performance liquid chromatography, confirmed that the spectral changes were not the result of conversion of substrate to product (Supplementary Figures S2B and S4). Unlike complexes of S_−__1__−__2_, only minor spectral changes were observed for hFEN1-S_−__8__−__9_ ± Ca^2+^, where the adjacent 2APs are firmly embedded in the substrate duplex (Figure 2F). Furthermore, electrophoretic mobility shift assay showed that all the substrates were at least 95% bound by hFEN1 under the conditions used (Supplementary Figure S2B and C). Therefore, we have spectral evidence of protein- and metal-ion-dependent conformational change of the substrate, analogous to that of product complex and indicative of unpairing of the substrate.

### Time-resolved fluorescence of 2AP single-strands, products and substrates

More detailed information about the nature of DNA conformational changes brought about by hFEN1-Ca^2+^ and mutants in the presence of metal ions was provided by time-resolved fluorescence spectroscopy. F or CF strands containing single 2AP substitutions were created. Where necessary, these were hybridized to template DNAs to form substrates S_+1_, S_−__1_, S_−__9_ and products, P_−__1_ and Q_+1_ (numbering denotes the site of 2AP modification, Figure 1A and B, Supplementary Figure S1) for analysis by time-resolved fluorescence spectroscopy.

**Figure 3.**
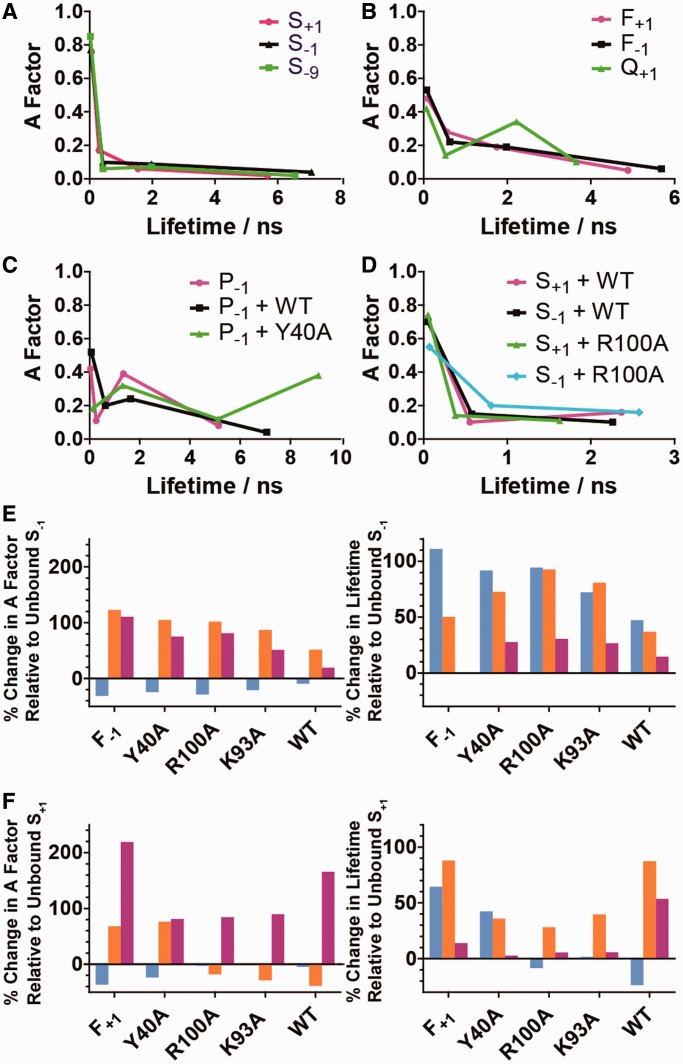
Graphical representation of the fluorescence decay parameters of 2AP-containing DNAs and hFEN1 WT- and mutant-DNA complexes. All spectra were recorded in Ca^2+^ containing buffers. (**A–D**) show plots of fractional amplitude (A factor) versus lifetime for unbound DNA constructs and selected complexes: (A) unbound S_+1_ (orange), S_−1_ (blue) and S_−9_ (green); (B) unbound F_+1_ (magenta), F_−1_ (black) and Q_+1_ (green); (C) unbound P_−1_ (magenta) and complexes of P_−1_ (black) and Y40A (green) with WT hFEN-1; (D) complexes of S_+1_ with WT (magenta) and R100A (green) and of S_−1_ with WT (black) and R100A (cyan). (**E**) and (**F**) show the percentage change in the fractional amplitudes (left) and corresponding fluorescence lifetime components (right) of each double-stranded substrate, S_−1_ (E) and S_+1_ (F), on conversion to the corresponding single strand or formation of the indicated protein complexes; τ_1_ and A_1_ cyan, τ_2_ and A_2_ orange, τ_3_ and A_3_ magenta Full sequences are shown in Supplementary Figure S1.

Initial fluorescence studies focused on the characterization of substrates and products in the absence of hFEN1. As seen previously, the fluorescence decays of the 2AP-containing DNAs were described by four lifetime components (τ_1__−__4_), reflecting the existence of a variety of conformational states (Figure 3A, Supplementary Table S1A) in which 2AP experiences different quenching efficiencies (11–15). Quenching is used here to mean the reduction in fluorescence lifetime (increase in non-radiative decay rate) as a result of intermolecular interaction. Earlier studies indicate that the shortest lifetime component (ca. 50 ps) corresponds to 2AP that is well stacked in the duplex and subject to rapid, fluorescence quenching, primarily due to inter-base electron transfer. The longest component of ca. 10 ns results from extra-helical 2AP (free from quenching), whereas two intermediate lifetimes are attributed to partially stacked forms. Thus, the magnitude of the lifetime reveals the nature of microenvironment of the 2AP (extent of stacking). The fractional amplitudes (A factors) of the four components (A_1__−__4_) indicate the fraction of the emitting population with a given lifetime and correspond to the relative populations of the various conformational states.

The fluorescence decay parameters of S_+1_, S_−__1_ and S_−__9_ are summarized graphically in Figure 3A as a plot of A factor versus lifetime, and the values are given in Supplementary Table S1A. They are all typical of well-formed duplex structures in which the vast majority of the 2AP population displays a short lifetime (τ_1_), characteristic of a highly stacked conformation. Interestingly, although the population of this rapidly quenched state is greatest (A_1_ = 85%) when 2AP is embedded well within the duplex (S_−__9_), it remains almost as high (ca. 75%) in the double-flap substrates with 2AP at the duplex termini (S_+1_ and S_−__1_). The maintenance of the terminal bases of duplex in an optimally stacked state within the double flaps is presumably a consequence of coaxial stacking of the two-way junction. This contrasts with measurements of simple duplex termini or forked DNAs, which show much greater amounts of unpairing at duplex ends (10). By analogy, a more heterogeneous conformational population than seen in the substrates would be expected for the 5′-phosphorylated product P_−__1_, with a 5′-terminal 2AP, as here the 3′-flap is not complementary to template DNA, reducing coaxial stacking. Indeed, in P_−__1_, 2AP was less well stacked than in the intact substrate with a large fraction of the population (ca. 60%) in weakly stacked states (Figure 3C, Supplementary Table S1A).

The fluorescence decay of 2AP is not sensitive to hydrogen-bonding interactions *per se*; thus, 2AP is not a direct probe of base pairing. However, the perturbation of base-stacking interactions that occurs as a result of local unpairing of the duplex gives rise to characteristic changes in the fluorescence decay parameters of 2AP, as we have shown previously in a systematic comparison of single strands and duplexes (15). This is evident here in the decay parameters of ss F_−__1_ and F_+1_ (Figure 3B, Supplementary Table S1A), when compared with those of the corresponding duplexes, as illustrated in Figure 3A and Supplementary Table S1A. Compared with the duplex, there was a sizeable transfer of population from the highly stacked state (decrease in A_1_) to states with sub-optimal stacking (increase in A_2_ and A_3_). There was also a substantial lengthening of decay times τ_1_ and τ_2_, indicative of a weakening of stacking interactions in the single strands. The population of the longest lifetime component remains small (only a few percent) in the single strands, indicating that the propensity for complete destacking of 2AP from its neighboring bases (i.e. base flipping) is no greater than in the duplex. When 2AP was the terminal 3′-nucleotide in ss product Q_+1_ and less constrained than intrastrand 2AP in ss F_+1_ and F_−__1_, there was an even more marked increase in occupancy of poorly stacked states (Figure 3B, Supplementary Table S1A).

The changes in the 2AP decay parameters that accompany the transition from duplex to single strand (i.e. duplex unpairing) can be represented schematically by the percentage change in lifetimes and A factors of components 1–3 in the single strand relative to the values in the duplex, as illustrated in Figure 3E, for F_−__1_ (ss) compared with S_−__1_ (ds), and in Figure 3F, for F_+1_ (ss) compared with S_+1_ (ds). (Component 4 has been omitted for simplicity because, as noted earlier in the text, it constitutes a small fraction of the emitting population in both single strands and duplexes).

### Unpaired hFEN1-product complexes detected by time-resolved fluorescence

To study the fluorescence decay, a WT hFEN1-Ca^2+^-P_−__1_ complex was formed, analogous to the P_−__1__−__2_ complex that provided evidence of unpairing by low energy CD. Surprisingly, a greater proportion of 2AP was rapidly quenched in hFEN1-Ca^2+^-P_−__1_ compared to the P_−__1_ in the unbound state (increase in A_1_ and A_2_, decrease in A_3_ and A_4_) as shown in Figure 3C and Supplementary Table S1B. We rationalized that when bound to WT hFEN1, fluorescence of 2AP in P_−__1_ is quenched by Y40, observed to stack on the unpaired −1 nucleotide in the crystal structure (Figure 1D). Previously, stacking of 2AP with tyrosine residues was shown to result in efficient charge-transfer quenching (13,14). To test this hypothesis, we prepared a Y40A-P_−__1_-Ca^2+^ complex and observed that quenching of 2AP in P_−__1_ was dramatically reduced (Figure 3C, Supplementary Table S1B). In the absence of Y40, ca. 40% of 2AP decayed with a lifetime of 9 ns, characteristic of an unquenched extrahelical environment. Nevertheless, there is also evidence that 2AP occupies intrahelical states in Y40A-P_−__1_-Ca^2+^; 18% of the 2AP still decays with a short lifetime (τ_1_) implying that a portion of the population remains well stacked on its nearest neighbor, although this is substantially reduced compared with the unbound duplex. This is in accord with the conclusions of low energy CD measurements of Y40A-Ca^2+^-P_−__1__−__2_.

### Unpairing of substrate by mutated hFEN1s monitored by time-resolved fluorescence

When Y40A, K93A and R100A complexes were formed with substrate S_−__1_ in the presence of Ca^2+^ ions, the fluorescence decays and associated parameters resembled those of the unbound ss flap F_−__1_ (Supplementary Tables S1A and C). This is illustrated in Figure 3E, where the changes in the decay parameters on binding of S_−__1_ by the mutated enzymes can be seen to closely resemble the changes that accompanied the transition from duplex to single strand. Thus, the 2AP in all these complexes sees a local environment that resembles a single strand, implying that both the −1 nucleotide and the adjacent +1 nucleotide are unpaired, in line with the conclusions of exciton coupling measurements. However, in enzyme-bound S_−__1_, the population of well-stacked states (A_1_) is somewhat greater than in ssF_−__1_ and the population of poorly stacked states (A_3_) somewhat less, suggesting an equilibrium between unpaired and paired conformations. It is notable that the decay parameters of 2AP in S_−__1_ are not sensitive to the presence of Y40 in the mutated complexes (the decay parameters of all three complexes are almost identical), implying that the −1 nucleotide does not stack with this tyrosine residue in the R93A or R100A substrate complexes.

As shown in Figure 3F (and Supplementary Tables S1A and C), the decay of S_+1_ also showed a clear shift toward that of an unpaired structure on binding to Y40A-Ca^2+^; there was a significant decrease in A_1_ (from 0.75 to 0.58) and corresponding increases in A_2_ (from 0.17 to 0.3) and A_3_ (from 0.06 to 0.10), together with increases in τ_1_ (from 60 ps to 80 ps) and τ_2_ (from 300 ps to 400 ps), in comparison with the unbound substrate. However, S_+1_ showed a different response when the substrate was bound to R100A or K93A, suggesting that 2AP in the +1 position may be affected by interaction with Y40. Indeed, in these complexes, there was enhanced quenching of 2AP compared with the Y40A complex (increase in A_1_ and decrease in τ_1_) reminiscent of that seen in the WT-P_−__1_ product complex (see earlier in the text). In crystal structures of hFEN1 with substrates that lack a 5′-phosphate, the DNA is seen to remain base-paired, with the +1 nucleotide positioned to stack with Y40 (Supplementary Figure S5A and B) (6). Therefore, the enhanced quenching seen in R100A-Ca^2+^ and K93A-Ca^2+^ S_+1_ complexes can be attributed to interaction of the +1 nucleotide with Y40, which is again suggestive of an equilibrium between unpaired and paired conformations.

### Unpaired hFEN1-substrate complexes detected by time-resolved fluorescence

When WT hFEN1 was bound to S_−__9_ in the presence of Ca^2+^ ions, no alteration of the decay parameters with respect to the free substrate was observed (Supplementary Table S1C). In contrast, the decay parameters of the WT- Ca^2+^-S_+1_ complex were altered with respect to free S_+1_ and resembled those of S_+1_ bound to K93A and R100A, with the quenching effect of Y40 clearly apparent (Figure 3D and F; Supplementary Table S3A). However, the values of A_3_ and τ_3_ were greater than in the mutated complexes, signifying greater disruption of interbase stacking; this increase may be the consequence of distortion required to stack the −1 base with Y40 (see later in the text). It is particularly significant that when S_−__1_ was bound to WT hFEN1, its decay parameters were different from those of all three mutated complexes and closely resembled those of the WT hFEN1-Ca^2+^-S_+1_ complex (Figure 3F, Supplementary Table S1C). This is emphasized in Figure 3D, where the mutated complexes are exemplified by R100A (the decay parameters of S_−__1_ in the three mutated complexes are almost identical). In complex with the WT protein, 2AP in each position in the substrate, +1 and −1, sees a similar microenvironment; both residues are quenched by stacking with Y40, presumably as a consequence of a paired-unpaired equilibrium. It is only in the WT complex that the −1 nucleotide of substrate appears able to stack with Y40, implying the necessity of residues R100 and K93 to form this particular unpaired state in substantial quantities.

## DISCUSSION

The necessity for double nucleotide unpairing of 5′-nuclease superfamily substrates has been inferred from biochemical studies and structural studies, but not yet observed (3–7). Our spectroscopic studies using 2AP-containing substrates provide the first direct evidence that the prototypical 5′-nuclease, hFEN1, can facilitate the unpairing of nucleotides surrounding the scissile phosphate diester bond. Our studies also reveal the components of the 5′-nuclease active site required for unpairing DNAs. An essential prerequisite for unpairing of both substrates and products is the presence of active site bound divalent metal ions, whose absence prevents unpairing in complexes with both DNAs. It has been noted previously that two (or more) metal ion active sites are associated with nucleases where DNA distortion is linked to active-site metal ion positioning of the scissile phosphate diester to afford specificity (18). The 5′-nuclease mechanism is an example of this phenomenon.

Surprisingly, 5′-nuclease superfamily strictly conserved (K93, R100) and semi-conserved residues (Y40), whose mutations are severely detrimental to catalysis in a range of family members (6,7,19), are not required to effect unpairing of substrates per se. Instead, active-site divalent metal ions are sufficient, although there is clear local variation in the respective conformation of unpaired nucleotides observed by CD with mutated proteins Y40A and R100A. Interestingly, in hFEN1 complexes with active-site metals, but devoid of DNAs, the region of the protein bearing K93 and R100 is disordered (α4-α5), as is the top portion of the Y40 helix (α2) (3,6,20,21). Moreover, residues of α2 pack against α5 when these regions are structured. As DNA conformational change can occur without these conserved residues, the possibility that nucleotide unpairing promotes arrangement of these regions of the protein to catalyze reaction is attractive. Such a mechanism would associate assembly of constituents of the active site with the presence of correctly unpaired substrate, thereby linking catalysis to specificity.

In contrast to the two unpaired nucleotides of substrate, both the conserved basic residues K93 and R100 are required to stabilize the single nucleotide unpaired product complex. Although some unpaired product can be formed with Y40A-Ca^2+^, the absence of Y40 appears to alter the partition between paired and unpaired forms of product or the respective orientation of the −1 and −2 nucleotides. As K93, R100 and Y40 are key to efficient catalysis, it seems likely that the requirements for unpaired product conformation most closely mimic those of double nucleotide unpaired substrate that is correctly positioned to react.

Time-resolved fluorescence identifies a unique equilibrium in WT hFEN1-substrate complexes, where interaction of Y40 with both the −1 and +1 nucleobases either side of the scissile bond can be detected. Although we cannot rule out an intermediate orientation of substrate that is unpaired, but positioned in such a way that allows Y40 to interact with the +1 base, the simplest explanation of this phenomenon is that paired substrate DNAs are present alongside their unpaired counterparts. The detected interaction of Y40 with the −1 nucleotide of the unpaired substrate, unique to the WT protein, requires the presence of the basic residues of the active site. This indicates that the characteristic 5′-nuclease superfamily active-site residues cooperate to produce what is likely to be close to the reactive conformation and would be consistent with an unpaired substrate inducing assembly of key active-site residues.

FEN1 substrate conformational change is reminiscent of the unpairing observed when DNA polymerases transfer the 3′-terminus of substrates to the 3′-exonuclease domain in editing mode. This substrate unpairing has also been studied using low energy CD of 2AP dimer substrates and time-resolved fluorescence measurements involving both 2AP and dyes (22–25). In DNA polymerases, occupation of the divalent metal ion binding 3′-exonuclease site is increased when the duplex has 3′-mismatched ends, although this site is still occupied to a lesser extent by base-paired DNAs, including their 2AP-T analogue. With the static double-flap substrate alone, the target duplex is not in a state that is substantially frayed, and neither is this state induced when bound to protein without divalent metal ions, despite the protein bending the substrate by 100° and breaking the coaxial stacking of the double flap. In contrast to polymerase editing, the FEN1 superfamily is tasked with recognizing base-paired ends (that abut DNA junctions), presumably to identify and preserve perfect duplex during replication or restoration of the genome. Nevertheless, similar overall principles, despite subtle differences, apply to both 5′-nucleases and 3′-exonucleases; a duplex binding site separated from an active site requires substrates to unpair for hydrolysis to occur. In both cases, this affords reaction specificity for duplex ends that can be unpaired, whereas the particular strand of duplex that undergoes reaction is selected by the precise juxtaposition of duplex binding and active sites.

These principles may also apply more widely to other structure- and strand-specific nucleases. Unrelated 5′-nucleases, such as the toroidal lambda nuclease, appear likely to achieve strand selectivity by unpairing ends (26), and DNA co-crystal structures of the 3′-nuclease Mre11 also suggest substrate unpairing is necessary for contact between active-site metal ions and the target phosphate diester (27). Degradative exoribonucleases (28–30), including some that conserve the FEN active site (3), seem to use unpairing of ends to deal with regions of RNA secondary structure and preserve reaction polarity. Thus, duplex-end unpairing, used by FEN1 family members to locate their reaction site and ensure reaction is specific for the 5′-strand, appears likely to be a common feature of both DNA- and RNA-acting structure- and strand-specific nucleases. The combination of low-energy CD and time-resolved fluorescence of 2AP-containing substrates will be a valuable methodology to explore the possible generality of this phenomenon.

## SUPPLEMENTARY DATA

Supplementary Data are available at NAR Online.

## FUNDING

Biotechnology and Biological Sciences Research Council [BB/J00300X to J.A.G.]; Marie Curie International Incoming Fellowship [project number 254386 to L.D.F.]. A.B., J.E. and E.J. thank the EPSRC and the University of Sheffield for studentships, and L.M. is grateful for the award of a CSC Scholarship. Funding for open access charge: BBSRC.

*Conflict of interest statement*. None declared.

## Supplementary Material

Supplementary Data
