# Supplementary material for: Observation of unpaired substrate DNA in the flap endonuclease-1 active site
Source: Nucleic Acids Res. 2013 Aug 23;41(21):9839–47. doi: 10.1093/nar/gkt737 (PMC3834815; doi:10.1093/nar/gkt737)
Supplement: Supplementary Data [file supp_41_21_9839__index.html]

Observation of unpaired substrate DNA in the flap endonuclease-1 active site — Observation of unpaired substrate DNA in the flap endonuclease-1 active site — Observation of unpaired substrate DNA in the flap endonuclease-1 active site — Supplementary Data 

# Observation of unpaired substrate DNA in the flap endonuclease-1 active site

## Supplementary Data

files

**Files in this Data Supplement:**

- Supplementary Data - docx file
