# Supplementary material for: Observation of unpaired substrate DNA in the flap endonuclease-1 active site
Source: Nucleic Acids Res. 2013 Aug 23;41(21):9839–47. doi: 10.1093/nar/gkt737 (PMC3834815; doi:10.1093/nar/gkt737)
Supplement: Supplementary Data [file supp_gkt737_nar-01146-h-2013-File005.docx]

**Supplementary Information Finger *et al.***

**Supplementary Figure S1**

**S1A**

**

**

**S1B**

| **Oligo** | **Sequence** |
| --- | --- |
| **F_+1_** (ss) | 5’-TTT TT(**2AP**) AGA GGC AGA GTG-3’ |
| **F_-1_** (ss) | 5’-TTT TTA(**2AP**)GA GGC AGA GTG-3’ |
| **F_-9_** (ss) | 5’-TTT TTA AGA GGC AG(**2AP**) GTG-3’ |
| **CF_-1_** (ss) | 5’-(**P**)(**2AP**)G AGG CAG AGT G-3’ |
| **Q_+1_** (ss) | 5’-TTT TT(**2AP**)-3’ |
| **T_A_** | 5’-CAC TCT GCC TCT TGA CGG TGA AAC CGT CC-3’ |
| **CF_-1-2_** (ss) | 5’-(**P**)(**2AP**)(**2AP**)A GGC AGA GTG-3’ |
| **F_-1-2_** (ss) | 5’-TTT TTG (**2AP**)(**2AP**)A GGC AGA GTG-3’ |
| **F_-8-9_** (ss) | 5’-TTT TTA AGA GGC G(**2AP**)(**2AP**) GTC-3’ |
| **HO-CF_-1-2_** (ss) | 5’-(**HO**)(**2AP**)(**2AP**)A GGC AGA GTG-3’ |
| **T_B_** | 5’-CAC TCT GCC TTT CGA CAG CGA AGC TGT CC-3’ |
| **T_C_** | 5’-CAC TTC GCC TCT TGA CAG CGA AGC TGT CC-3’ |

**S1C**

| **Construct** | **Oligonucleotide composition** |
| --- | --- |
| **P_-1_** (ds) | CF_-1_ + T_A_ |
| **P_-1-2_** (ds) | CF_-1-2_ + T_B_ |
| **HO-P_-1-2_** (ds) | HO-CF_-1-2_ + T_B_ |
|  | |
| **S_+1_** (ds) | F_+1_ + T_A_ |
| **S_-1_** (ds) | F_-1_ + T_A_ |
| **S_-1-2_** (ds) | F_-1-2_ + T_B_ |
|  | |
| **S_-9_** (ds) | F_-9_ + T_A_ |
| **S_-8-9_** (ds) | F_-8-9_ + T_C_ |

**Figure S1. Oligonucleotides and Substrate and Product Constructs (A)** Schematic representation of oligonucleotide constructs with abbreviations shown and positions of 2AP indicated by the red A. (**B)** Sequences of individual oligonucleotides used. (**2AP**): 2-aminopurine, (**P**): 5’-phosphate and (**HO**): 5’-hydroxyl. The position of the 2AP is numbered relative to the scissile phosphate and indicated by the subscript (See Figure 1B main text). Single stranded (ss) oligos are indicated. (**C)** The double stranded (ds) product and substrate constructs in Figure S1A were prepared by annealing the indicated template (T) and flap strand (F or CF) oligonucleotides (Figure S1B) in a ratio of 1.1:1, respectively, in folding buffer (100 mM KCl, 50mM HEPES pH 7.5). Concentrations of oligonucleotides were determined using extinction coefficients derived from the OligoAnalyzer® Tool (<http://eu.idtdna.com/analyzer/Applications>).

**Supplementary Figure S2**


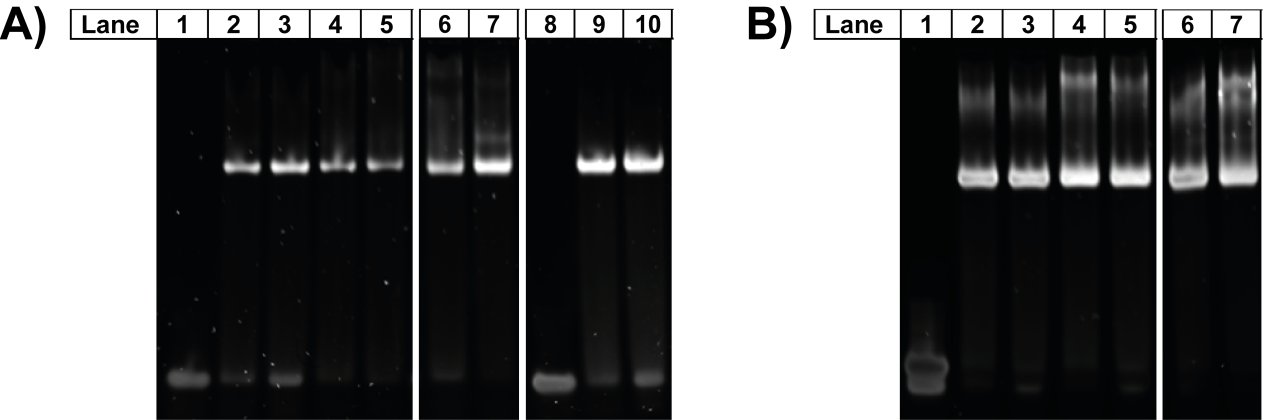


**Figure S2.** **Electrophoretic mobility shift assays** (EMSA) of product and substrate heteroduplexes with WT and mutant hFEN1s under conditions analogous to CD experiments. Final enzyme and substrate concentrations were 12.5 µM and 10 µM respectively, in buffer (110 mM KCl, 55 mM, HEPES pH=7.5, 1 mM DTT with either 10mM CaCl_2_ alone or 10mM CaCl_2_ and 40mM EDTA). DNA was visualized using SYBR® Green and a ChemiDoc^TM^ Imaging System (BioRad). **(A)** Lane 1: P_-1-2_ (Ca^2+^), Lane 2: P_-1-2_ + WThFEN1 (Ca^2+^), Lane 3: P_-1-2_ + WThFEN1 (EDTA), Lane 4: P_-1-2_ + Y40A (Ca^2+^), Lane 5: P_-1-2_ + Y40A (EDTA), Lane 6: P_-1-2_ + R100A (Ca^2+^), Lane 7: P_-1-2_ + R100A (EDTA), Lane 8: HO-P_-1-2_ (Ca^2+^). Lane 9: HO-P_-1-2_ + WThFEN1 (EDTA), Lane 10: HO-P_-1-2_ + WThFEN1 (Ca^2+^). All samples were electrophoresed on the same gel. **(B)** Lane 1: S_-1-2_ (Ca^2+^), Lane 2: S_-1-2_ + WThFEN1 (Ca^2+^), Lane 3: S_-1-2_ + WThFEN1 (EDTA), Lane 4: S_-1-2_ + Y40A (Ca^2+^), Lane 5: S_-1-2_ + Y40A (EDTA), Lane 6: S_-1-2_ + R100A (Ca^2+^), Lane 7: S_-1-2_ + R100A (EDTA) . Quantitation of the gels in panels A and B indicates that >95% of the DNA is in complex with the enzyme.

**Supplementary Figure S3**

**
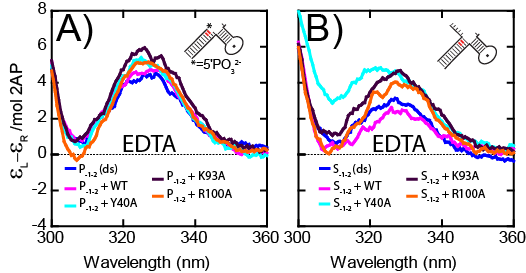
**

**Figure S3**. **Low energy CD spectra** of the P_-1-2_ **(A)** or S_-1-2_ **(B**) alone (blue) and in complex with either WT hFEN1 (magenta), Y40A (cyan), K93A (purple) or R100A (orange) in buffer containing 10 mM Ca^2+^ and **25 mM EDTA.** The same samples were analysed with no EDTA and results are shown in Figures 2C and 2E main text. **(A)** Spectra of P_-1-2_ alone and in complex with WT and mutant hFEN1 showing that the spectra are similar in the presence of excess EDTA. **(B)** Spectra of S_-1-2_ alone and in complex with WT and mutant hFEN1. With mutated hFEN1s an increase in signal at 330 nm is observed (with respect to the unbound DNA) upon complex formation in EDTA buffer; these contrast with the decreases observed in buffer containing Ca^2+^ alone (see Figure 2 main text).

**Supplementary Figure S4**


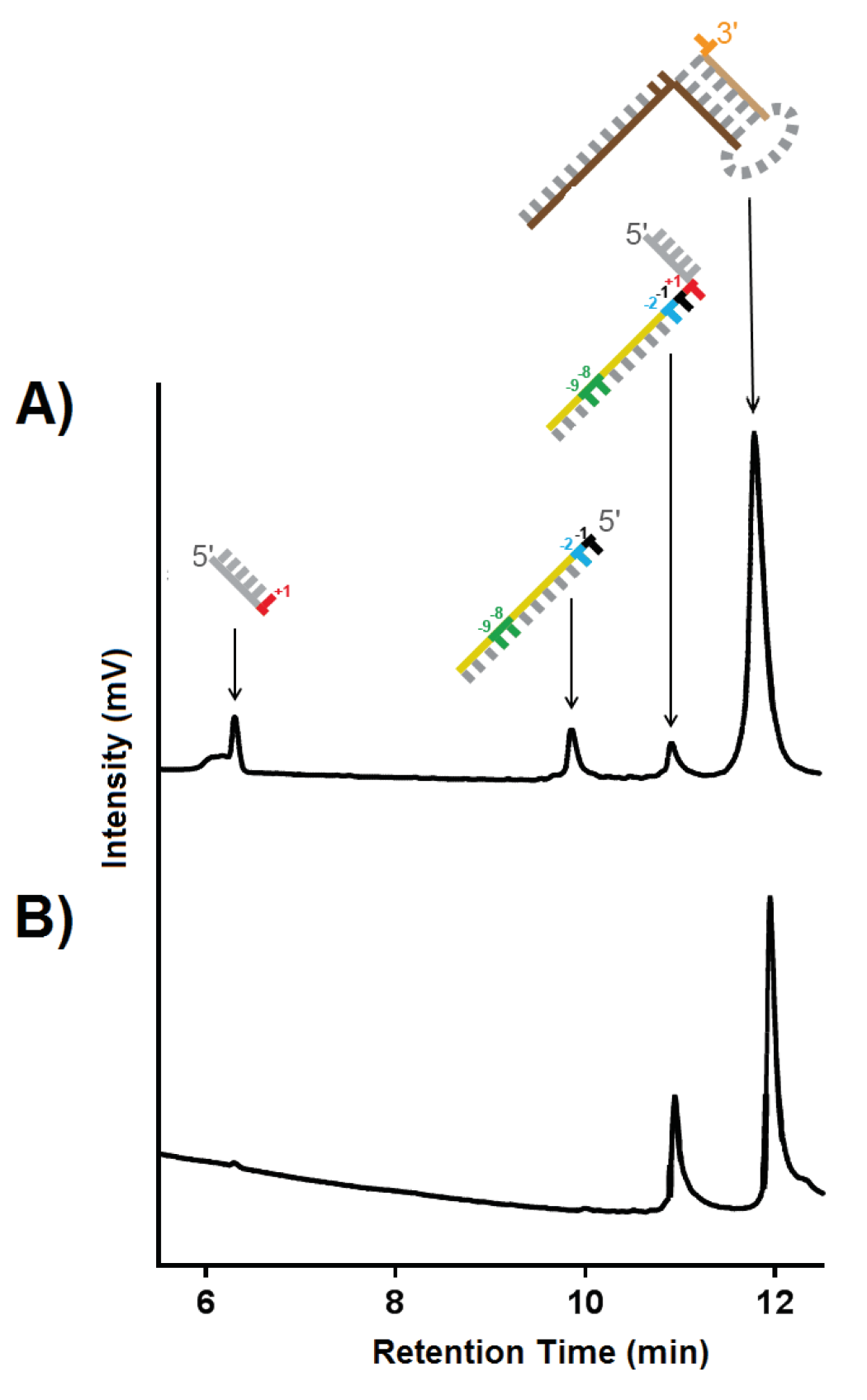


**Figure S4.** **Analysis for potential reaction of hFEN1-Ca^2+^-S complexes.** Denaturing HPLC chromatogram (UV) of **A)** an aliquot of a mock CD sample containing WThFEN1 and S_-1-2_ in the presence of Mg^2+^ and **B)** an aliquot of the CD sample containing WThFEN1 and S_-1-2_ in the presence of Ca^2+^. Prior to analysis, an aliquot of each sample was diluted in 8 M Urea and 80 mM EDTA to a final concentration of 100 nM. Denaturing HPLC retention times for the two products P and Q (as in Figure 1) and the uncleaved flap and template strands are 6.31 min, 9.88 min, 11.32 min and 12.25 min, respectively. Denaturing HPLC was perfromed on a WAVE® system, Transgenomic, UK equipped with UV detector using tetrabutyl ammonium bromide containing buffers and a linear gradient of acetonitrile as described ([1](#_ENREF_1)).

**Supplementary Figure S5**


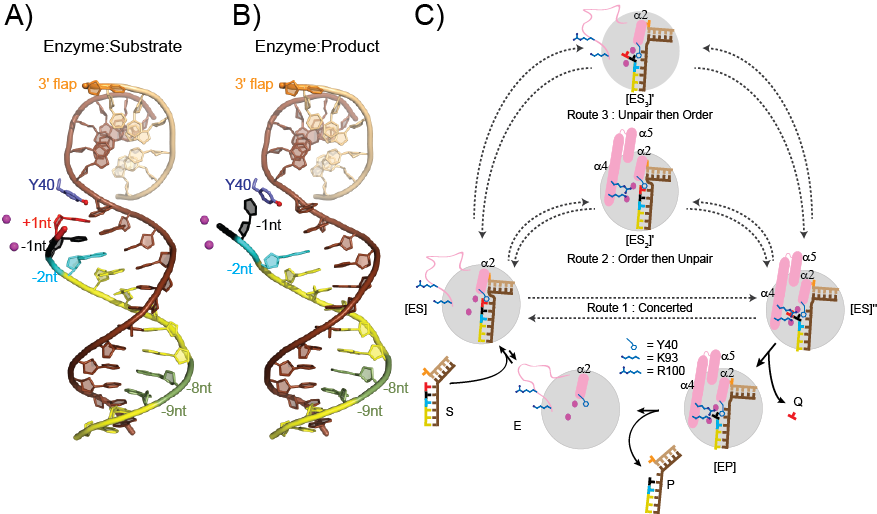


**Figure S5.** Cartoon representation of the DNA in the hFEN1 **(A)** enzyme-substrate (3Q8L) and **(B)** enzyme-product complexes (3Q8K) highlighting the positions of the nucleotides (nts) referred to in Figure 1C. Active site metal ions are shown as purple spheres. The protein is not shown except for the side chain of Y40. Y40 interacts with the 5'-face of the +1 nt and 3'-face of -1 nt in the substrate and product complexes, respectively.

**Supplementary Table S1**

Fluorescence lifetimes (τ_i_) and their fractional amplitudes (A_i_) for unbound substrates and products and their complexes with wild type and mutated hFEN1s.

All decays were recorded in 50 mM HEPES pH 7.5, 10 mM CaCl_2_, 100 mM KCl, and 1 mM DTT unless indicated.

**A** Fluorescence lifetimes (τ_i_) and their fractional amplitudes (A_i_) for free substrates, products and flap strands as defined in Figure S1.


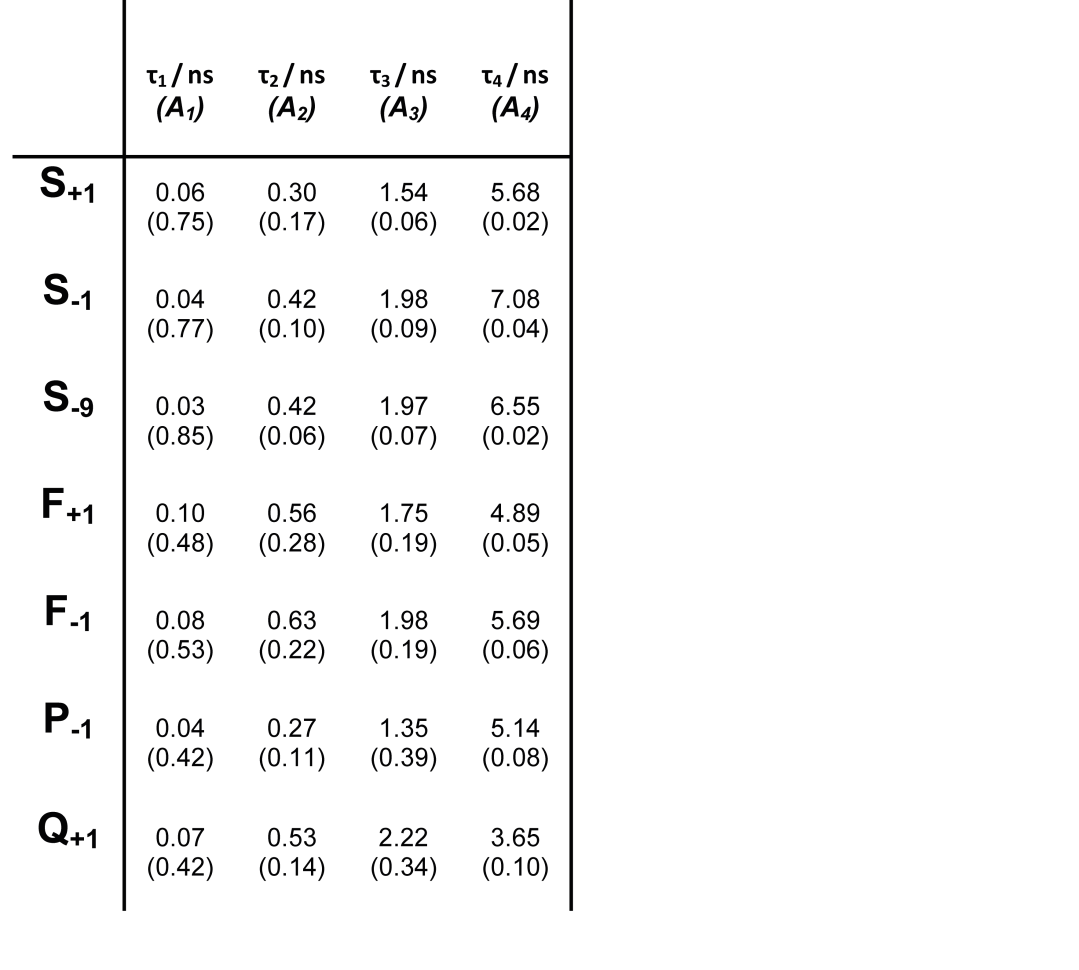


**B**

Fluorescence lifetimes (τ_i_) and their fractional amplitudes (A_i_) for free products and enzyme-product complexes as defined in Figure S1. Q_+1_ was generated from addition of 10 mM Mg^2+^ to a complex of WT hFEN1 and S_+1_ in buffer without Ca^2+^ and produced very similar parameters to those of synthetic Q_+1_ in Ca^2+^ shown in Table S1A. All other decays were recorded in the presence of Ca^2+^ ions using synthetic DNA constructs.


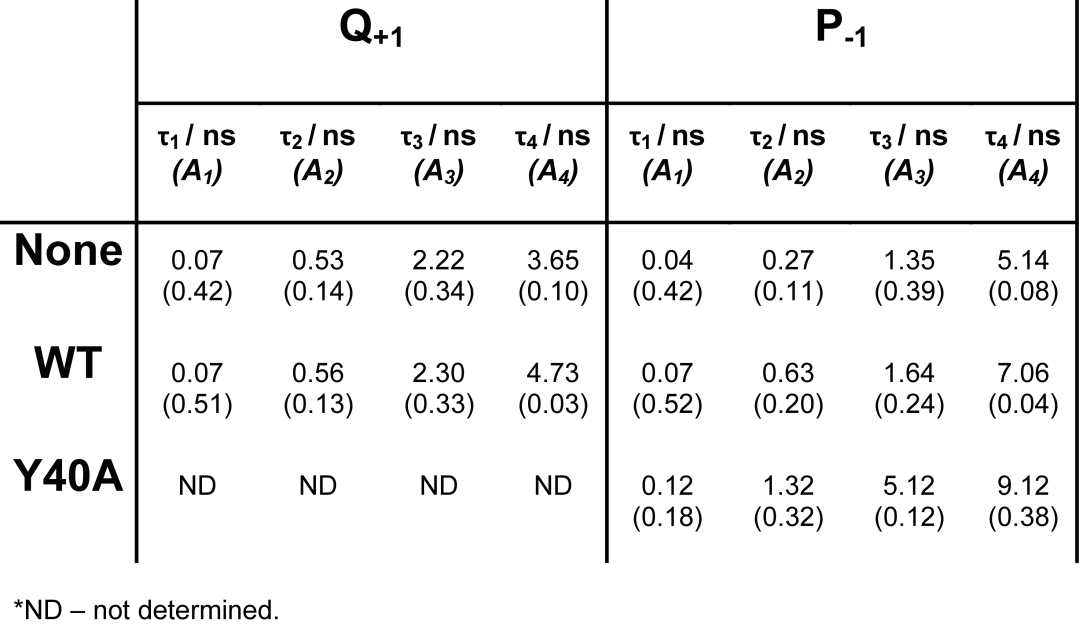


**C**

Fluorescence lifetimes (τ_i_) and their fractional amplitudes (A_i_) for free substrates and enzyme-substrate complexes as defined in Figure S1.


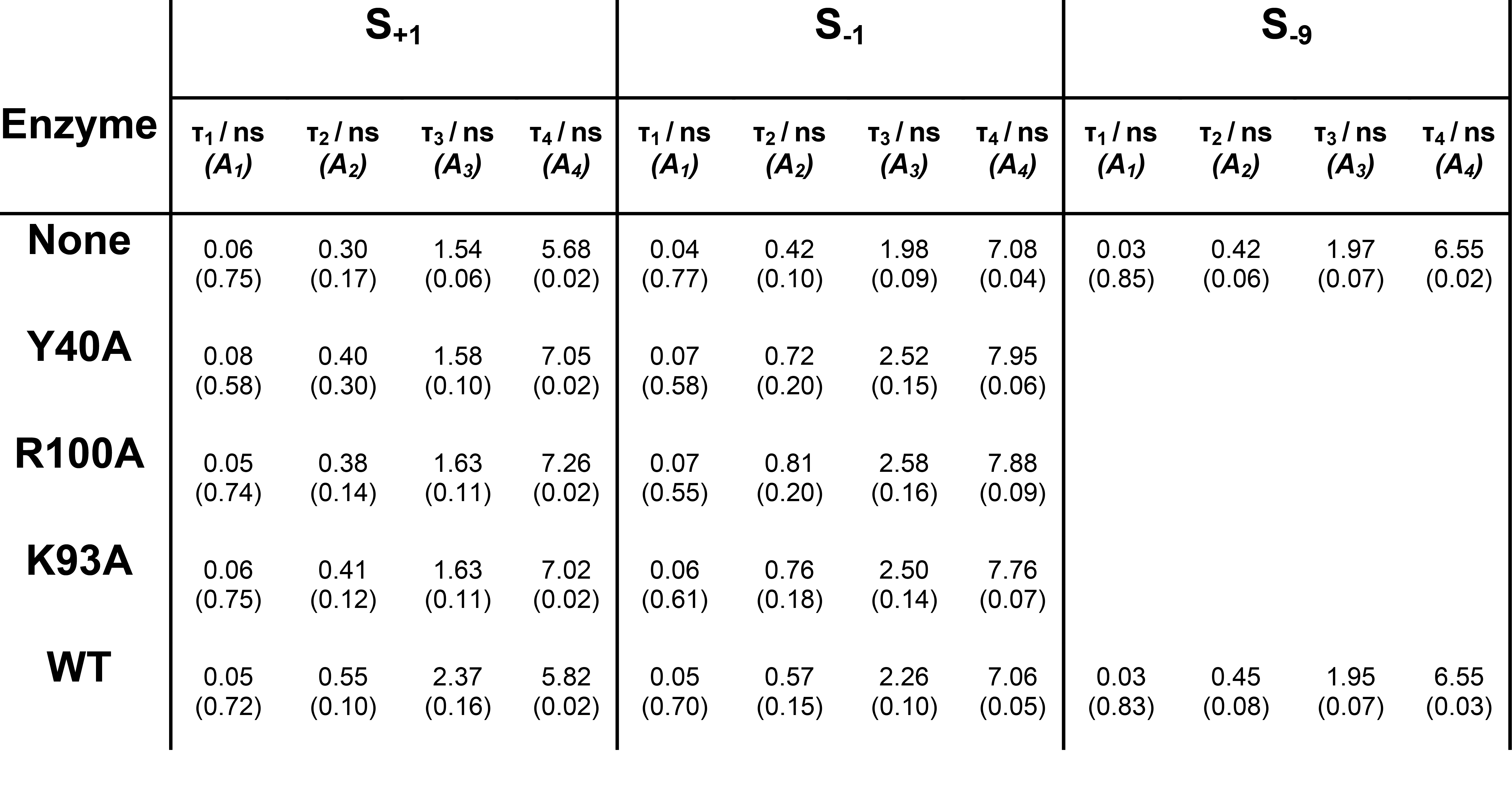


**Supplementary References**

1. Finger, L.D., Blanchard, S.M., Theimer, C.A., Sengerova, B., Singh, P., Chavez, V., Liu, F., Grasby, J.A. and Shen, B. (2009) The 3'-flap pocket of human flap endonuclease 1 is critical for substrate binding and catalysis. *J. Biol. Chem.*, **284**, 22184-22194.
